# NADCdb: A Joint Transcriptomic Database for Non-AIDS-Defining Cancer Research in HIV-Positive Individuals

**DOI:** 10.3390/ijms27031169

**Published:** 2026-01-23

**Authors:** Jiajia Xuan, Chunhua Xiao, Runhao Luo, Yonglei Luo, Qing-Yu He, Wanting Liu

**Affiliations:** MOE Key Laboratory of Tumor Molecular Biology and State Key Laboratory of Bioactive Molecules and Druggability Assessment, Institute of Life and Health Engineering, College of Life Science and Technology, Jinan University, Guangzhou 510632, China; xuanjj@jnu.edu.cn (J.X.); xch2023@stu.jnu.edu.cn (C.X.); luorunhao@stu2022.jnu.edu.cn (R.L.); yonglei1115@stu.jnu.edu.cn (Y.L.)

**Keywords:** non-AIDS-defining cancer (NADC), people living with HIV (PLWH), cancer, biomarkers, joint analysis strategy

## Abstract

Non-AIDS-defining cancers (NADCs) have emerged as an increasingly prominent cause of non-AIDS-related morbidity and mortality among people living with HIV (PLWH). However, the scarcity of NADC clinical samples, compounded by privacy and security constraints, continues to present formidable obstacles to advancing pathological and clinical investigations. In this study, we adopted a joint analysis strategy and deeply integrated and analyzed transcriptomic data from 12,486 PLWH and cancer patients to systematically identify potential key regulators for 23 NADCs. This effort culminated in NADCdb—a database specifically engineered for NADC pathological exploration, structured around three mechanistic frameworks rooted in the interplay of immunosuppression, chronic inflammation, carcinogenic viral infections, and HIV-derived oncogenic pathways. The “rNADC” module performed risk assessment by prioritizing genes with aberrant expression trajectories, deploying bidirectional stepwise regression coupled with logistic modeling to stratify the risks for 21 NADCs. The “dNADC” module, synergized patients’ dysregulated genes with their regulatory networks, using Random Forest (RF) and Conditional Inference Trees (CITs) to identify pathogenic drivers of NADCs, with an accuracy exceeding 75% (in the external validation cohort, the prediction accuracy of the HIV-associated clear cell renal cell carcinoma model exceeded 90%). Meanwhile, “iPredict” identified 1905 key immune biomarkers for 16 NADCs based on the distinct immune statuses of patients. Importantly, we conducted multi-dimensional profiling of these key determinants, including in-depth functional annotations, phenotype correlations, protein–protein interaction (PPI) networks, TF-miRNA-target regulatory networks, and drug prediction, to deeply dissect their mechanistic roles in NADC pathogenesis. In summary, NADCdb serves as a novel, centralized resource that integrates data and provides analytical frameworks, offering fresh perspectives and a valuable platform for the scientific exploration of NADCs.

## 1. Introduction

HIV remains one of the most pressing global public health challenges, significantly compromising the health and quality of life of people living with HIV (PLWH) [1]. According to The Joint United Nations Programme on HIV/AIDS, approximately 40.8 million individuals were living with HIV by the end of 2024 [2]. Since the first reported case of HIV-1 infection, certain malignancies—termed AIDS-defining cancers (ADCs)—have been predominantly associated with PLWH, including Kaposi’s sarcoma, non-Hodgkin’s lymphoma, and primary central nervous system lymphoma [3,4]. The introduction and widespread use of highly active antiretroviral therapy (HAART) have led to a marked decline in ADC incidence, alongside an improved life expectancy and quality of life for PLWH. However, non-AIDS-defining cancers (NADCs) have emerged as a leading cause of non-AIDS-related morbidity and mortality in this population [1,5,6,7,8,9]. Studies indicate that PLWH face an elevated risk of mortality from 30 out of 40 NADC types—including lung, liver, and small intestine cancers—compared to the general population [1]. Consequently, understanding the evolving patterns of NADC is essential for effectively preventing and managing these malignancies and ensuring optimal outcomes in PLWH [10].

Recent studies have proposed several hypotheses to explain the mechanisms of NADC, with immunosuppression, chronic inflammation, oncogenic viral co-infections, and direct HIV-mediated carcinogenesis emerging as key contributing factors [11,12,13]. Persistent HIV infection induces chronic inflammation through dysregulated cytokine production (e.g., elevated IL-6 and TNF-α), fostering a pro-tumorigenic microenvironment that supports angiogenesis and cancer cell survival [14]. Simultaneously, the HIV-driven depletion of CD4^+^ T cells weakens immune surveillance, allowing nascent cancer cells to evade detection and elimination [15]. Additionally, many NADCs are associated with co-infections by established oncogenic viruses, such as the human papillomavirus (HPV) and hepatitis B/C viruses [16]. Notably, HIV itself may directly promote oncogenesis through multiple mechanisms, including synergistic interactions with other viruses, the disruption of cell cycle regulation, the inactivation of tumor suppressor genes, chromosomal instability via telomerase inhibition, impaired DNA repair, enhanced tumor angiogenesis, and the potentiation of exogenous carcinogens [17]. Previous studies have explored the occurrence and development mechanisms of NADC from the perspective of specific cancer types. For instance, Chen et al. utilized proteomic analysis to identify systemic crosstalk between interferon-related antiviral responses and oncogenic pathways in HIV-1-associated colorectal cancer (HACRC) [18]. Similarly, Wu et al. employed transcriptomic analyses to uncover an overrepresentation of mitotic pathways in HIV-1-associated lung cancer (HALC) [19], while Bao et al. investigated key pathways, including vascular endothelial growth factor-activated receptor and lipopolysaccharide receptor activities, through RNA-seq mining in HIV-1-associated renal cancer (HARC) [20]. Despite these advances, our understanding of NADC pathogenesis remains markedly incomplete. The field currently faces several major challenges and gaps: (i) Although over 30 NADC types have been linked to elevated morbidity and mortality, only a limited subset has been rigorously studied [1]. (ii) While multi-omics approaches offer powerful tools for comprehensively analyzing the dynamic mechanisms of biological systems in pathological states, omics-level investigations have thus far been restricted to only four NADC types, with a combined sample size of fewer than 100 cases [18,19,20,21,22]. (iii) There is a critical deficiency in systematic methodologies and platforms to support research into the mechanistic underpinnings of NADC development. Data scarcity remains a fundamental bottleneck in this field of research, stemming from multiple practical constraints. The unique complexities of HIV infection pose significant operational challenges in acquiring clinical samples of NADCs, including biosafety concerns during sample collection and stringent patient privacy protections. Additionally, the historically limited research focus on these cancers has resulted in an insufficient accumulation of multi-omics data. These combined factors have led to a critical shortage of high-quality omics data pertaining to NADCs, substantially impeding systematic and in-depth investigations into their pathogenesis. However, a joint analysis approach offers a viable solution by enabling the synthesis and examination of omics data from both HIV and cancer studies. For example, through a joint analysis approach, Zhou et al. identified six biomarkers, including AURKA, in rheumatoid arthritis and major depressive disorder [23]; Jiang et al. uncovered immune-related hub genes in asthma and depression [24]; and Zhang et al. elucidated key genes within the oxidative stress pathway in pain–depression comorbidity [25]. Leveraging the established biological links between HIV and cancer, and in light of the current scarcity of NADC samples, we employed a joint analysis strategy. This approach involves the comprehensive integration and systematic exploration of existing omics data from HIV infection and related cancers to identify pivotal biological factors that may critically influence NADC development and progression. This methodology offers a novel perspective for advancing our understanding of the pathological mechanisms underlying non-AIDS-defining cancers.

**Figure 1:**
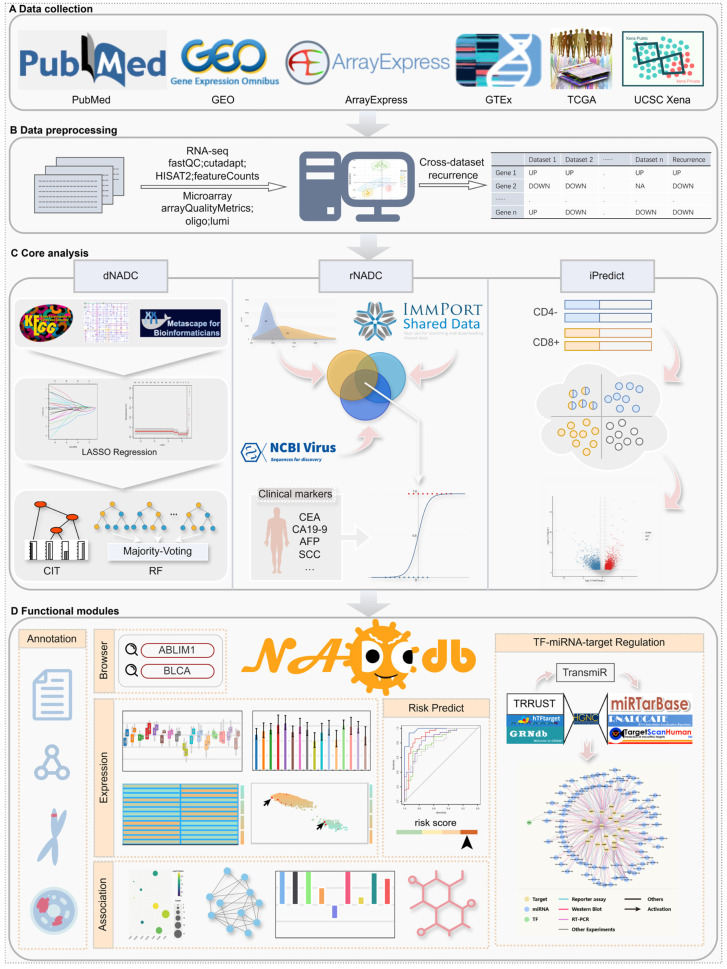
The workflow of NADCdb. (**A**) Data collection. (**B**) Standardized data preprocessing pipelines. (**C**) Core modules for biomarker discovery and risk assessment. (**D**) Functional modules within NADCdb that facilitate intuitive browsing and exploration of key regulatory factors potentially involved in NADC pathogenesis.

Here, we introduce NADCdb (http://bioinformaticsscience.cn/nadcdb, accessed on 1 January 2026), a publicly available database dedicated to NADCs, employing a joint analysis strategy to systematically assess NADC risk in PLWH and identify key biomarkers across 23 NADC types (Figure 1). NADCdb integrates six core analytical modules: (i) “rNADC” consolidates 314 NADC-specific factors to construct a risk assessment model based on immunosuppression, chronic inflammation, and clinical biomarkers, supplemented with an interactive “rNADC-tool” that allows users to upload independent datasets for personalized risk evaluation; (ii) “dNADC” identifies diagnostic biomarkers by mapping dysregulated genes shared between PLWH and cancer patients onto the Kyoto Encyclopedia of Genes and Genomes (KEGG) [26] regulatory pathways, achieving >90% prediction accuracy in an external validation cohort of HIV-associated clear cell renal cell carcinoma and >75% accuracy for other HIV-associated cancers in internal testing; (iii) “iPredict” innovatively screens 1905 potential immune biomarkers across 16 NADCs using HIV-specific immune signatures (CD4 decline and CD8 elevation) [27,28,29]; (iv)”Regulation” elucidates genome–transcriptome interactions in NADCs by reconstructing transcription factor (TF)-miRNA regulatory networks of key factors; (v) multi-dimensional analyses—including functional enrichment, WGCNA [30], the protein–protein interaction (PPI) network, and drug prediction—further decode NADC regulatory mechanisms; and (vi) two intuitive modules, “Gene” and “Cancer”, facilitate the user-friendly exploration of regulatory relationships between key biomarkers and NADCs via gene- or cancer-specific queries. This comprehensive resource provides novel insights into NADC pathogenesis and serves as a valuable tool for researchers investigating HIV-associated oncogenesis.

## 2. Results

### 2.1. The Comprehensive Exploration of NADC Diagnostic Biomarkers by “dNADC”

**Figure 2:**
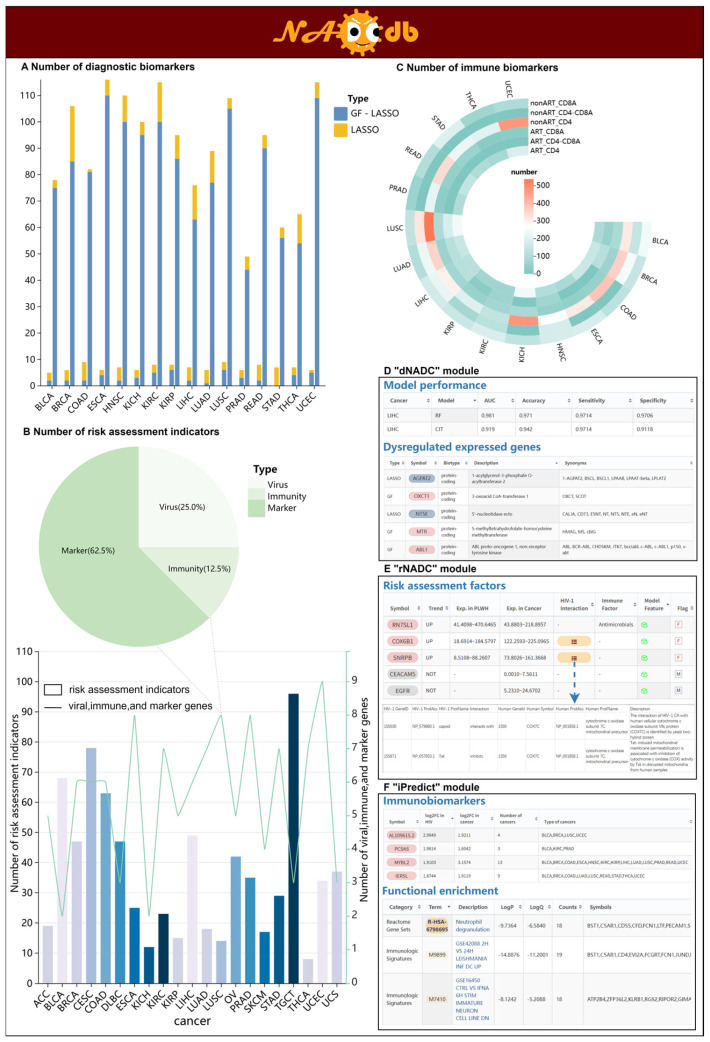
The three core models in NADCdb. (**A**) Number of diagnostic biomarkers identified by “dNADC” in 16 NADCs. “GF” refers to gene feature, indicating that the gene is used as a feature directly for modeling. (**B**) Number of risk assessment indicators identified by “rNADC” in 21 NADCs. (**C**) Number of immune biomarkers recognized by “iPredict” in 16 NADCs. (**D**) The performance of the “dNADC” model across different types of NADCs and its identified diagnostic factors. (**E**) “rNADC” recognizes risk factors for different NADCs based on immunosuppression, chronic inflammation, and clinical markers. (**F**) “iPredict” identifies subgroup-specific immune signatures according to immunological status.

To discern HIV-associated diagnostic biomarkers potentially driving NADC pathogenesis, we first determined key genetic features by screening for dysregulated genes shared between 237 PLWH (Table S1) and 7716 cancer patients (Sample sizes of 16 types of cancer range from 91 to 1194, Table S2), along with identifying HIV-associated differentially expressed genes in upstream regulatory cascades in KEGG pathways of NADCs. These features were subsequently integrated into Random Forest (RF) and Conditional Inference Tree (CIT) algorithms to construct distinct “dNADC” models for individual NADC types. The “dNADC” models demonstrated a diagnostic accuracy exceeding 75% in 68.75% (11/16) of NADCs, with a particularly notable performance observed in the internal test sets for renal chromophobe carcinoma (KICH) and uterine corpus endometrial carcinoma (UCEC), where accuracy rates surpassed 90%. The “dNADC” interface enables users to query the performance metrics of each “dNADC” model, including Area Under the Curve (AUC), accuracy, sensitivity, and specificity. Significantly, this module allows the systematic exploration of diagnostic biomarkers recognized by individual “dNADC” models, with the number of characterized markers ranging from 1 to 116 across models (Figure 2A,D, Table 1). Notably, we optimized diagnostic markers for all 16 NADCs using the Least Absolute Shrinkage and Selection Operator (LASSO), generating more precise molecular evidence that further advances our understanding of NADC pathogenesis in PLWH.

### 2.2. Risk Assessment of NADC Development in PLWH Using “rNADC”

Systematically evaluating the risk of PLWH developing various NADCs is crucial for early monitoring and intervention in patients, which can reduce NADC incidence and improve survival rates. Considering the impact of HIV-related adverse effects such as immune suppression and chronic inflammation on cancer development, we integrated 1793 relevant genes from public platforms as candidate risk factors for assessing 21 NADC occurrences. By deciphering their gene expression profiles, we systematically identified 314 key factors showing consistent upward or downward expression trends in both 100 PLWH and 9786 cancer patients (Figure 2B,E). Given that dysregulation in clinical biomarkers represents another critical factor influencing cancer progression, we combined the above key factors with clinical markers collected from PubMed to form the final risk assessment indicators for different NADCs (Table 2). Ultimately, the “rNADC” model was constructed by integrating bidirectional stepwise regression and logistic regression to evaluate the risk of PLWH developing distinct NADCs. The “rNADC” webpage comprehensively displays risk factors for different NADCs, including their abnormal trends, expression levels, and factor types in both PLWH and cancer patients. In the “rNADC” module, users can view gene annotations of risk factors through the “Symbol” button and explore their relationships with viral infection via the “HIV-1 interaction” button.

### 2.3. Deciphering the Landscape of Immune Signatures Across Distinct Immune Statuses in Patients by “iPredict”

Given that HIV primarily targets the human immune system, leading to a decline in CD4 and an increase in CD8 during the chronic stage [27,28,29], we, respectively, categorized 356 PLWH and 7716 cancer patients into three groups based on distinct immune statuses, including “CD4-” (decreased CD4), “CD8+” (increased CD8), and “CD4-CD8+” (decreased CD4 accompanied by increased CD8). Given the established feasibility of mapping tissue-specific gene expression to blood profiles, as demonstrated by Basu et al. [31], we next identified potential immunobiomarkers. For each group, we detected genes with shared dysregulation profiles in both PLWH and cancer patients as potential immunobiomarkers, yielding a total of 1905 markers across 16 NADC types (Figure 2C,F; Table 3 and Table 4). These immune biomarkers from distinct groups were systematically stored in the “iPredict” module, providing their gene annotations and dysregulation profiles, along with statistical documentation of their occurrence frequencies across various cancers. This multi-dimensional presentation may reveal common pathogenic mechanisms among different NADCs. Furthermore, to facilitate a comprehensive assessment of the significance and effect size of the dysregulated state, NADCdb also provides three key metrics for each biomarker: effect size (log2FC), adjusted *p*-value (FDR), and Cohen’s d value for cross-feature comparison.

### 2.4. Performing Multi-Dimensional Annotations of Key Factors

**Figure 3:**
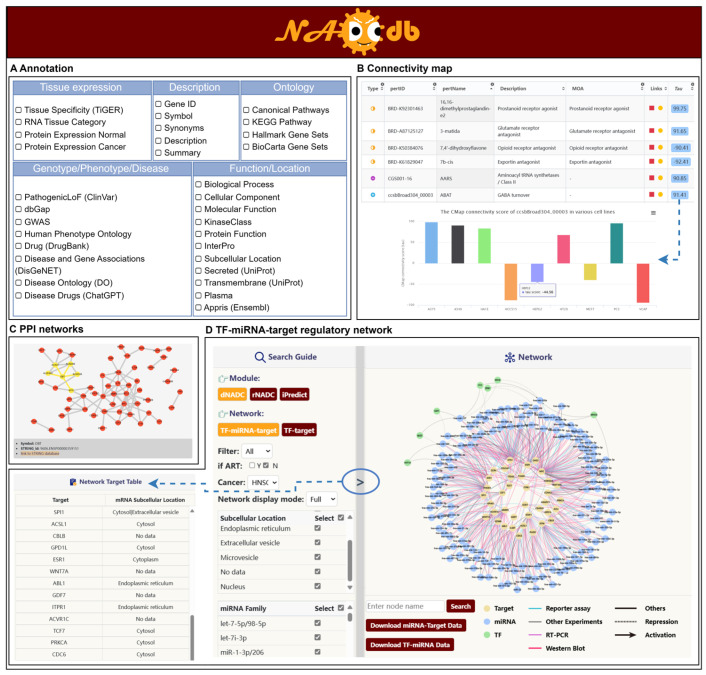
Multi-dimensional analysis of key factors with potential regulatory roles in NADC. (**A**) Gene annotations encompassing descriptive, ontological, functional, locational, phenotypic, and disease-related information. (**B**) PPI network. (**C**) CMap analysis. (**D**) TF-miRNA-Target regulatory network in “Regulation” module.

To further elucidate the potential molecular mechanisms underlying NADC development in PLWH, we conducted comprehensive annotations of all key factors characterized by the aforementioned three models. These annotations encompass descriptive, ontological, functional, subcellular locational, phenotypic, and disease-related information (Figure 3A). For NADC subtype-specific critical biomarkers identified through aforementioned models, we also performed functional enrichment analysis and constructed PPI networks to decode the complex upstream and downstream regulatory networks (Figure 3B). Users can dynamically retrieve associated data from authoritative databases such as AmiGO [32] and STRING [33] by clicking Term IDs in tables or network nodes. Significantly, integrated analysis via the Connectivity Map (CMap) [34] identified a set of candidate therapeutic small molecules, providing multi-dimensional and reliable evidence for further understanding the progression mechanisms and therapeutic strategies of NADCs (Figure 3C). Within the interactive platform, users can acquire a CMap connectivity score matrix of critical factor–compound pairs across different cell lines using the Tau button, and seamlessly access compound activity heatmaps and comprehensive pharmacological profiles in external databases through the “Link” button.

### 2.5. Construction of TF-miRNA-Target Regulatory Networks

Transcription factors (TFs) and microRNAs (miRNAs) serve as pivotal regulators in transcriptional and post-transcriptional gene expression, respectively. Their synergistic interactions and regulatory networks hold significant value in deciphering disease mechanisms [35,36]. To systematically elucidate the potential molecular mechanisms of key factors in the pathogenesis of NADCs, we integrated 1,326,241 TF-target regulatory pairs, 10,699 miRNA-target regulatory interactions, and 2957 TF-miRNA interaction pairs from public databases. Through a systematic analysis of interactions between these regulatory elements and key molecules recognized by three core NADC modules, we constructed a comprehensive regulatory network comprising 4091 TF-target regulatory pairs, 2043 miRNA-target regulatory associations, and 1033 TF-miRNA-target regulatory trios. To enable multi-dimensional visualization, the network was modularized based on model type, NADC subtype, regulator category (TF/miRNA), subcellular localization/family classification of targets, and miRNA family, and was integrated into an interactive “Regulation” analytical module (Figure 3D). In the “Regulation” interface, users can dynamically filter and generate specific TF-target or TF-miRNA-target regulatory networks. To address visualization challenges in complex networks, the platform incorporates a “co-regulation” filtering function, which optimizes network topology by retaining core TFs and miRNAs that regulate multiple targets. This systematic dissection of the regulatory network provides critical theoretical insights into the multi-layered molecular mechanisms driving NADC initiation and progression.

### 2.6. Browse Modules

**Figure 4:**
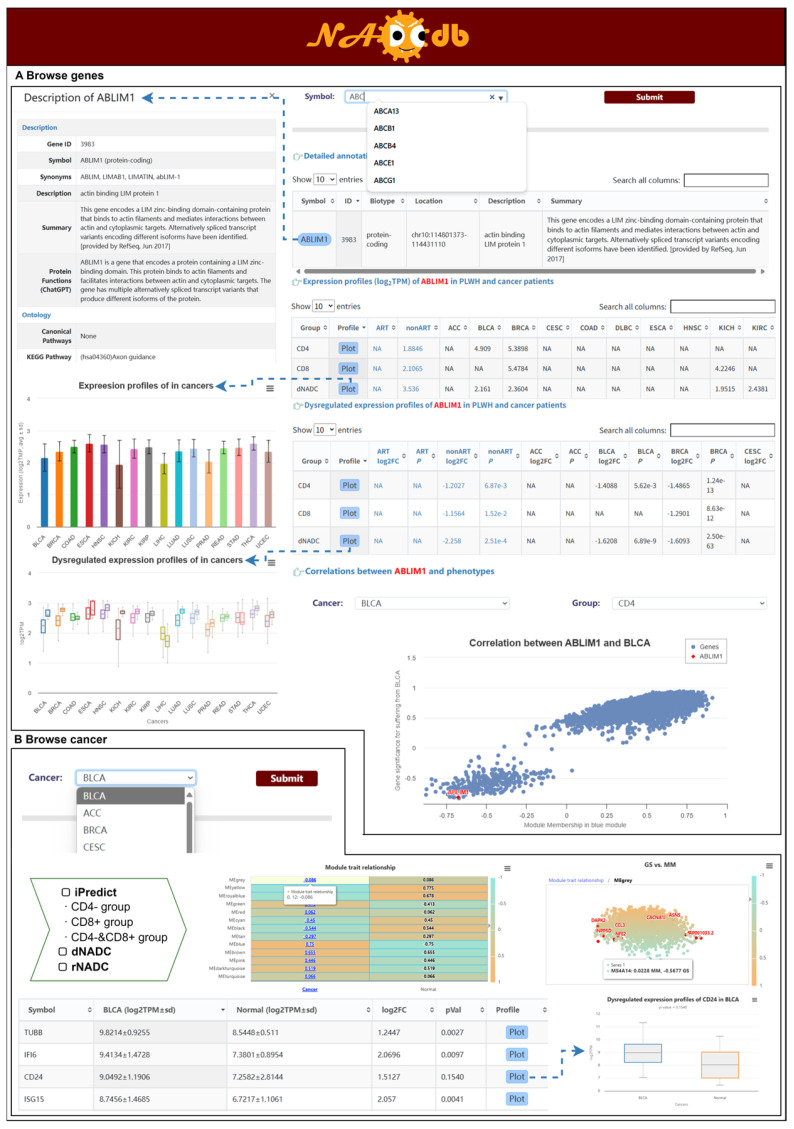
Data browser modules. (**A**) Gene-based search. Returns expression profiles of specified genes in PLWH and cancer patients, along with gene–phenotype correlations. (**B**) Cancer-specific search. Retrieves key genes for designated NADC types across different analytical models, including their expression profiles and gene–phenotype correlations.

In addition to the modules mentioned above, we have also constructed two user-friendly browsing modules, the “Gene” module and the “Cancer” module, to facilitate searching, browsing, visualizing, and downloading key NADC factors of interest. The “Gene” module enables users to retrieve key gene features of interest. It provides gene annotations, expression profiles, and differential expression profiles in PLWH and cancer patients, along with WGCNA results that show gene–phenotype correlations (Figure 4A). These indicators help users quickly identify and prioritize genes of interest. The “Cancer” module allows users to retrieve the expression profiles and dysregulated expression profiles of critical factors from multiple models, including “rNADC”, “dNADC”, and “iPredict”, using cancer names. It also provides effect sizes and the cancer differential expression status for genes within each selected cancer, enabling a more targeted screening and interpretation of candidate genes. All models incorporate interactive module–trait association diagrams, where clicking any numerical value visualizes detailed gene–module correlations and gene–phenotype correlations. These results, presented in tabular or graphical formats, can be clicked on to access detailed information or download images (Figure 4B).

### 2.7. Tool Modules

**Figure 5:**
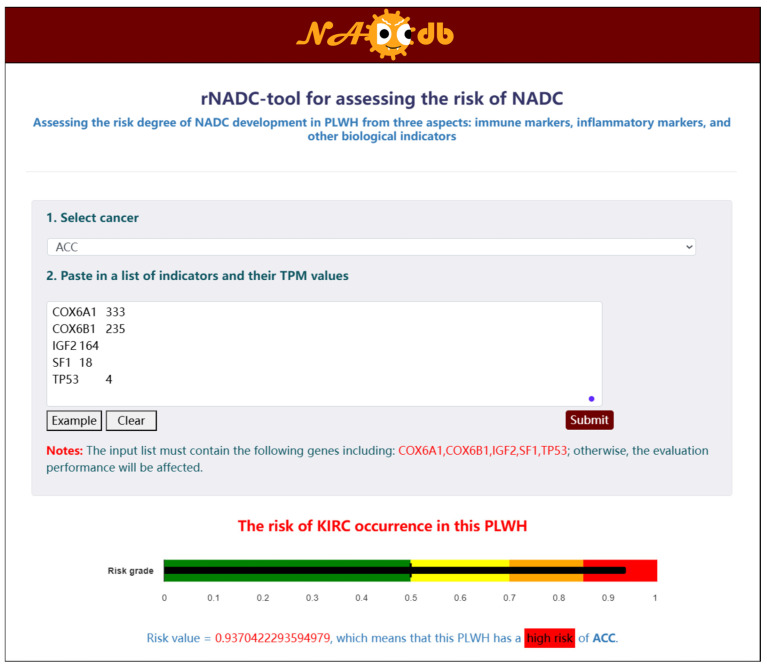
Tool module based on “rNADC” model for assessing the risk of PLWH developing NADCs.

To fully demonstrate the functional capabilities of NADCdb and enhance user service, we developed a user-friendly interactive “rNADC-tool” module based on the “rNADC” model for assessing the risk of PLWH developing 21 NADC subtypes. Within the interface of this tool, users must first select a target NADC subtype for prediction, then input the specified feature names and corresponding expression values in the required format. Upon submission, the system displays the calculated risk score and corresponding risk stratification at the bottom of the page (Figure 5).

### 2.8. Case Study: Identification of Key Genes and Potential Diagnostic Biomarkers for HARC

**Figure 6:**
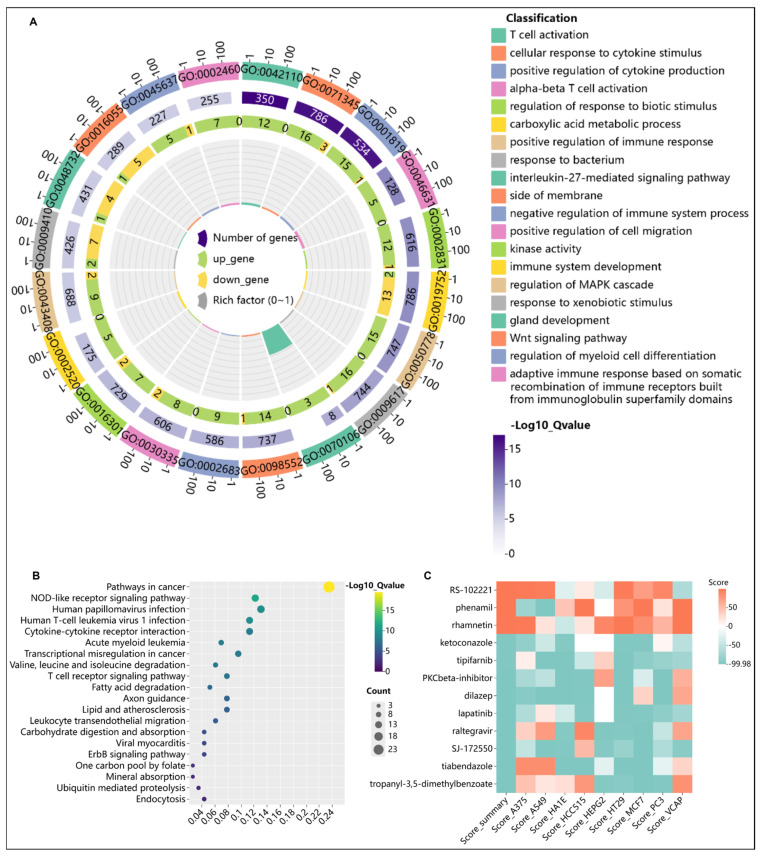
(**A**) Gene ontology enrichment analysis of 115 key HARC-associated genes. (**B**) KEGG pathway enrichment analysis of 115 key HARC-associated genes. (**C**) CMap analysis for potential therapeutic compound identification.

Through “dNADC” analysis, renal carcinoma differential genes underwent KEGG pathway enrichment and were integrated with upstream regulatory genes, followed by an intersection with HIV differential genes. This identified 115 key HARC genes (Figure S1A,B) that exhibit an abnormal expression in HIV patients and may function as regulatory or cancer-associated genes potentially contributing to HARC pathogenesis. To elucidate the functional roles and mechanistic contributions of these genes, functional enrichment and KEGG analyses were performed. Gene ontology (GO) enrichment analysis revealed a significant enrichment in T cell activation and cellular response to cytokine stimulus (Figure 6A,B), both of which represent core pathological features of HIV infection. Meanwhile, KEGG analysis highlighted pathways in cancer as the most prominent, followed by multiple immune- or viral-related pathways, suggesting their potential direct involvement in the progression from viral infection to tumor development. These findings delineate a preliminary molecular framework wherein immune dysregulation serves as a critical link between HIV infection and cancer development.

To investigate potential therapeutic small-molecule compounds for HARC patients, the CMap database was used to predict compounds capable of reversing HARC-related pathogenic gene expression patterns. Twelve small-molecule compounds with scores above the 99.5th percentile were identified as particularly promising therapeutic candidates for HARC (Figure 6C). Notably, compounds including Lapatinib and Ketoconazole may exert therapeutic effects by inhibiting oncogenic ErbB/MAPK signaling, regulating protein stability via ubiquitination pathways or enhancing immune responses.

**Figure 7:**
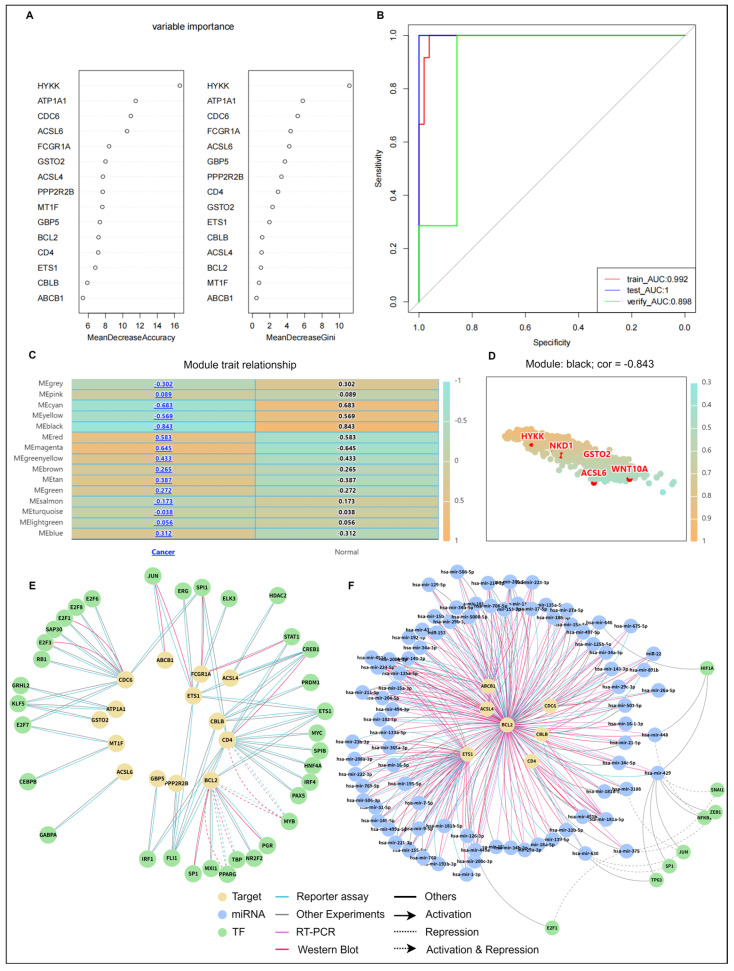
Establishing a diagnostic model for HARC using machine learning and uncovering associated regulatory networks. (**A**) Random Forest-based variable importance ranking of candidate biomarkers. (**B**) ROC curve analysis validating the HARC diagnostic model. (**C**) Module–trait correlation heatmap from WGCNA. (**D**) Scatter plot of diagnostic biomarkers versus module membership in the most relevant module. (**E**) Regulatory network of predicted TFs targeting diagnostic biomarkers. (**F**) Regulatory network of predicted miRNAs targeting diagnostic biomarkers.

To refine diagnostic biomarkers, LASSO regression selected 15 hub genes as candidate biomarkers for developing an optimal diagnostic model (Figure S2A,B). Figure 7A presents the ranking results of 15 genes based on two metrics (Mean Decrease Accuracy and Mean Decrease Gini) from the RF algorithm. The results demonstrate that the genes HYKK, ATP1A1, and CDC6 consistently ranked at the top under both evaluation criteria, indicating their critical discriminatory contribution to constructing the HARC diagnostic model. Notably, the immune function-related genes CD4 and GBP5 also showed high importance rankings (Figure 7A). Model discriminatory performance was evaluated using ROC curves (Figure 7B), achieving an AUC of 0.898 in the HARC validation set, demonstrating an excellent discriminatory capability for HARC.

Furthermore, NADCdb incorporates WGCNA to pinpoint gene modules correlated with disease traits. A correlation heatmap identified the MEblack module as having the strongest negative correlation (r = −0.843) with KIRC (Figure 7C). Strikingly, HYKK—the highest-ranked gene in the Random Forest analysis (Figure 7A)—was a member of this key module, thereby strengthening its candidacy as a central biomarker through convergent evidence from two independent analytical methods.

The construction of a TF-mRNA-miRNA network enabled the prediction of target TFs and miRNAs for diagnostic biomarkers, providing deeper insights into gene regulatory mechanisms. This approach establishes a theoretical foundation for developing targeted therapies. Through the TF-miRNA-target model in the “Regulation” module, target miRNAs and their TFs for hub genes were identified, while TF-target selection predicted target TFs. Among the 15 hub genes, 14 were predicted to have 34 TFs (Figure 7E), while 7 genes were associated with 83 miRNAs (Figure 7F). Furthermore, seven of these miRNAs were predicted to be regulated by eight TFs. Detailed information is openly available through the NADCdb database.

## 3. Discussion

NADCs have become a predominant contributor to non-AIDS-related morbidity and mortality in PLWH, drawing significant attention in epidemiological research [1,5,6]. While several hypotheses have been proposed to explain NADC pathogenesis [11,12,13], its underlying mechanisms remain incompletely understood. Research progress has been hindered by challenges in obtaining clinical samples and the scarcity of relevant data. To address the aforementioned limitations, this study adopts a joint analysis strategy, combining HIV and cancer transcriptomic data to explore potential molecular regulatory mechanisms in NADC. The findings from 23 cancer types have been systematically integrated into NADCdb.

The scientific value of NADCdb lies in its focus as a “disease-, population-, and database-specific” resource, addressing a critical gap in bioinformatics tools for HIV-associated cancer research. Unlike general immuno-oncology databases such as The Cancer Immunome Atlas (TCIA) and TISIDB [37,38], NADCdb is tailored to the biological complexity of NADCs in the context of HIV infection. It pioneered a joint analysis strategy to systematically mine key biomarkers with potential regulatory roles in NADC development from 12,486 transcriptomic profiles of PLWH and cancer patients, thereby overcoming the bottleneck of scarce NADC data. Furthermore, it concurrently considers the interactions among viral expression, chronic immune activation, and tumorigenesis, directly addressing the unique core biological questions specific to HIV-NADC. Building on this foundation, NADCdb has developed three core models designed to assess NADC risk in PLWH, identify key drivers influencing NADC progression, and explore potential immune biomarkers for NADC. Significantly, NADCdb conducted multi-dimensional analyses of biomarkers screened across these models, including exhaustive functional annotations, expression profiles, PPI networks, TF-miRNA-target regulatory networks, phenotypic correlation assessments, and small-molecule targeted drug prediction, providing indispensable insights into the molecular mechanisms governing NADC pathogenesis.

Several methodological considerations warrant attention. First, the small-molecule associations inferred from the CMap database are computationally predicted and should be regarded as exploratory leads rather than clinically validated therapies. Second, the “rNADC-tool” represents a proof-of-concept model for NADC risk assessment; its clinical applicability requires validation through prospective studies and functional experiments. Third, to reduce bias from imbalanced sample sizes across datasets, a stringent differential expression threshold (|log2FC| > 1) was applied to large-scale cohorts. While this enhances signal robustness, it may exclude weaker but biologically relevant signals. Future studies using size-matched cohorts will help refine these findings.

A major limitation of the current study is the lack of large-scale external validation, owing to limited public data availability. Performance metrics based on internal validation may therefore be optimistic and should be interpreted as preliminary. The primary contribution of this work lies in proposing a research framework and a repository of candidate targets for HIV-associated cancers; clinical translation will require systematic evaluation in future prospective studies.

It should also be noted that shared differentially expressed genes between blood and tumor tissue are not necessarily specific to HIV infection. Systemic inflammatory responses induced by cancer, immune dysregulation related to HIV, and alterations within the tumor microenvironment may collectively shape the observed transcriptional profiles. Enriched pathways such as “T cell activation” and “cytokine–cytokine receptor interaction” likely reflect the compounded effects of HIV infection, tumor–immune remodeling, and potential co-infections (e.g., HPV, HTLV-1) (Figure 6A,B). The future integration of single-cell sequencing, multi-omics data, and functional assays will help disentangle these contributions.

Furthermore, the iPredict module is designed to explore systemic immune-driven molecular patterns that may transcend tissue boundaries, rather than to directly map blood-based CD4/CD8 expression to tissue. This approach provides computational support for the hypothesis that systemic immune dysregulation influences tumor progression, although causal mechanisms await validation using paired tissue–blood samples.

By focusing on PLWH with stable, chronic infections, this study controls for disease-stage heterogeneity but may limit generalizability to individuals in acute or AIDS phases. Future validation across broader HIV disease spectra will help assess the dynamic relevance of the identified biomarkers.

This study systematically delineates the molecular characteristics of HIV-associated tumors; however, the expression patterns of these features and their potential for clinical translation may be modulated by several external key factors. Growing evidence suggests that ART can promote the reconstruction of the immune environment, which serves as an important foundation for tumor immune surveillance and the formation of the tumor microenvironment [39,40,41]. Furthermore, co-infections (such as oncogenic viruses like HPV and HCV) and lifestyle factors (especially smoking) are not only potential confounders but also substantial effect modifiers [16]; they can independently or synergistically alter local and systemic immune landscapes, genomic stability, and treatment responses, thereby potentially affecting the stability and generalizability of the features identified in this study across diverse populations [42,43,44]. In the future, with sufficient research data, developing population-calibrated prediction models capable of integrating these multidimensional determinants will be crucial for achieving reliable risk stratification and guiding treatment decisions in real-world clinical settings.

NADCdb has been technically validated with an initial module-loading time of ~45 s and query response times under 1 s. To ensure the system’s continuous and reliable operation, we have established long-term monitoring mechanisms and provide open feedback channels (such as email) to promptly receive and address user suggestions and issue reports. The platform is fully compatible with mainstream browsers and their common versions (including Chrome 128.1.6541.23, Edge 143.0.3650.96, etc.) and features a dedicated data download page that supports the one-click export of relevant datasets and analysis results, facilitating local research and verification.

To ensure that the database remains a current and powerful resource for the research community, NADCdb is committed to an annual update cycle, with major releases planned each June. These versions (e.g., v1.0, v2.0) will be communicated via our official website. Our ongoing efforts will be dedicated to the systematic integration of novel multi-omics data, with a focus on transcriptomic and proteomic profiles from PLWH and cancer cohorts. A foremost task is to include rigorously validated datasets from NADC patients, which is crucial for the continuous improvement of our analytical models and the refinement of their predictive power. Meanwhile, we sincerely welcome researchers in this field to submit or share updated data with us at any time, so that we can further optimize the model and continuously improve its predictive performance and the reliability of the results.

In conclusion, NADCdb integrates extensive transcriptomic data with joint analytical frameworks to provide multidimensional resources for decoding NADC biology. It offers a platform to explore pathological mechanisms, advance precision diagnosis, and inform targeted therapeutic strategies for NADCs in PLWH.

## 4. Materials and Methods

### 4.1. Collection of HIV Datasets

Through the search terms “HIV RNA-seq” or “HIV microarray”, a total of 4908 samples were collected from the GEO and ArrayExpress [45,46]. The samples that were only infected with HIV were retained, while those with co-infections or other disease were discarded. All samples were divided into two groups: one receiving highly effective antiretroviral therapy (ART) and the other receiving no ART (non-ART). All subsequent analyses were performed separately for each group. Datasets with indeterminate information or concomitant medications beyond ART were deliberately excluded. Given that the progression of HIV infection in individuals generally encompasses the acute, chronic, and AIDS phases [47], and recognizing that the chronic phase is the most prolonged and the main period for the onset of NADCs [28], only samples from the chronic phase of HIV infection were retained. Microarray and RNA-seq data from whole blood and Peripheral Blood Mononuclear Cell (PBMC) samples of 205 non-ART cases, 147 ART cases, and a substantial cohort of 241 healthy control samples were ultimately utilized for analysis (Table S1).

### 4.2. Processing of HIV Microarray Datasets

For microarray datasets, different processing methods were selected according to manufacturer. The R package “arrayQualityMetrics (v3.56.0)” [48] was used to perform quality control and outlier removal. For Affymetrix microarrays, the R package “oligo (v1.64.1)” [49] was applied to obtain the P/A call of each probe set. The probe sets with “A” in more than 50% of samples in different groups are removed. The “rma” function was used to convert probe signal intensity into expression values. For Illumina microarrays, probes with detection *p* value < 0.01 in at least 25% of samples in different groups were retained. The “lumi (v2.52.0)” [50] package was applied to convert probe signal intensity into expression values. Finally, the filtered probe sets were annotated with the annotation files of each chip platform.

### 4.3. Processing of HIV RNA-Seq Datasets

The raw sequence reads were processed using a comprehensive pipeline. Initially, quality control was performed using FastQC (v0.12.1) and MultiQC (v1.17) [51]. The following were removed to obtain clean reads: (1) reads containing an adapter, (2) reads containing ploy-N, (3) low-quality reads from raw data, and (4) reads with a length of less than 20 nt after triming. Additionally, Q20, Q30, and GC content were calculated on clean data. All downstream analyses were performed on high-quality clean data. The cleaned reads were aligned to the reference genome using HISAT2 (v2.1.0) [52]. The aligned reads were then quantified using featureCounts from the Subread package (v2.0.6) [53]. The GRCh38.p13 reference genome and the corresponding annotations (v36) from GENCODE [54] were used for the above analyses.

### 4.4. Collection and Processing of Cancer Datasets

The 13,669 gene expression profiles for 33 cancer types, including Adrenocortical Carcinoma (ACC), Bladder Urothelial Carcinoma (BLCA), Breast Invasive Carcinoma (BRCA), Cervical Squamous Cell Carcinoma and Endocervical Adenocarcinoma (CESC), Cholangiocarcinoma (CHOL), Colon Adenocarcinoma (COAD), Diffuse Large B-cell Lymphoma (DLBC), Esophageal Carcinoma (ESCA), Glioblastoma Multiforme (GBM), Head and Neck Squamous Cell Carcinoma (HNSC), Kidney Chromophobe (KICH), Kidney Renal Clear Cell Carcinoma (KIRC), Kidney Renal Papillary Cell Carcinoma (KIRP), Acute Myeloid Leukemia (LAML), Brain Lower Grade Glioma (LGG), Liver Hepatocellular Carcinoma (LIHC), Lung Adenocarcinoma (LUAD), Lung Squamous Cell Carcinoma (LUSC), Mesothelioma (MESO), Ovarian Serous Cystadenocarcinoma (OV), Pancreatic Adenocarcinoma (PAAD), Pheochromocytoma and Paraganglioma (PCPG), Prostate Adenocarcinoma (PRAD), Rectum Adenocarcinoma (READ), Sarcoma (SARC), Skin Cutaneous Melanoma (SKCM), Stomach Adenocarcinoma (STAD), Testicular Germ Cell Tumors (TGCT), Thyroid Carcinoma (THCA), Thymoma (THYM), Uterine Corpus Endometrial Carcinoma (UCEC), Uterine Carcinosarcoma (UCS), and Uveal Melanoma (UVM), were obtained through the R package “TCGAbiolinks (v2.28.4)” [55] from The Cancer Genome Atlas (TCGA) [56] combined with manual downloads from the Genotype-Tissue Expression (GTEx) [57] and UCSC Xena [58] database. From these, 11,893 gene expression profiles across the 23 cancer types, each comprising at least 10 normal tissue samples and 10 matched adjacent normal tissue samples, were subjected to subsequent analyses (Table S2). In each dataset, genes with less than 10 counts in at least 50% of samples were excluded.

### 4.5. Differential Expression Analysis

The “limma (v3.56.2)” package [59] was used to identify differentially expressed genes (DEGs) individually within each HIV and cancer dataset. The *p*-value was adjusted based on the false discovery rate (FDR) correction method. For RNA-seq data, genes with adjusted *p*-value < 0.05 and |log2FC| > 1 were considered statistically significant. For microarray data, genes with adjusted *p*-value < 0.05 and |log2FC| > log2(1.5) were considered significantly dysregulated. Subsequently, the recurrence of each DEG across all datasets was tallied. Genes exhibiting a frequency greater than n/2 (where n represents the total number of datasets) were designated as the final set of robust, cross-validated DEGs.

### 4.6. Construction of the NADC Risk Assessment Models for PLWH

A total of 1793 immune-related genes and inflammatory factors were obtained from the Immunology Database and Analysis Portal website [60]. After extracting the expression matrix of immune-related genes and inflammatory factors in cancer and HIV datasets, respectively, the outliers were identified by the “boxplot.stats” function [61] and were replaced by average values. For each gene, we defined transcripts per million (TPM) in healthy controls as the interval x_1_~x_2_, covering 90% of the whole blood samples. If at least 90% of the samples in the disease group demonstrate a TPM greater than x_2_ (or less than x_1_), we labeled these genes with a trend mark “UP” (or “DOWN”). Genes showing the same trends in both HIV and cancer datasets were considered as key immunity genes or inflammatory factors.

By integrating HIV-1-human protein interaction data from the HIV-1, Human Protein Interaction Database [62], we identified predictive features for model training. Clinical biomarkers were subsequently incorporated to enhance model performance. The final risk assessment model of PLWH developing NADCs was constructed using bidirectional stepwise regression followed by logistic regression analysis.

### 4.7. Construction of NADC Diagnostic Models

The DEGs in cancer datasets enriched in KEGG pathways and the upstream genes from the regulatory pathways were intersected with DEGs in HIV whole blood datasets. The intersected genes were defined as HIV–cancer feature genes. Based on the TCGA paired samples, we randomly split the data into a training set and testing set at a 7:3 ratio to establish a diagnostic model for different NADCs separately. Two methods were used: the “randomForest (v4.7-1.1)” [63] R package for a Random Forest (RF) model and the “ctree” function in the “party (v1.3-15)” R package [64] for a Conditional Inference Tree (CIT) model. To optimize model performance, LASSO regression analysis [65] was performed to screen for HIV–cancer feature genes. The selected genes were then used to build more accurate diagnostic models. A 10-fold cross-validation was used to evaluate model performance. For KIRC, the GEO cohort GSE205204 [21] dataset was used as an external validation set to verify the accuracy of characteristic genes in the diagnostic model.

### 4.8. Prediction of NADC Immune Biomarkers

The HIV samples derived from PBMC and the cancer paired samples sourced from tissues were applied for the identification of immune biomarkers. These samples of HIV and cancers were separately stratified into three distinct dysregulated subgroups based on immune status: Group 1, with significantly downregulated CD4 (CD4-); Group 2, with markedly upregulated CD8 (CD8+); and Group 3, with significantly decreased CD4 accompanied by increased CD8 (CD4-CD8+). All remaining samples were classified as the control group. DEGs with the same dysregulated trend were considered as immune biomarkers.

### 4.9. TF-miRNA-Target Regulatory Network Construction

Transcription factor (TF)–target regulatory relationships were obtained from the TRRUST [66], hTFtarget [67], and GRNdb [68] databases. Experimentally validated miRNA-target interactions were extracted from miRTarBase [69], and only interactions supported by at least two of the following three types if evidence were retained: (1) Western blot, (2) RT-qPCR, and (3) reporter assay. Furthermore, target gene categorization was performed using human gene family information curated from the HUGO database [70]. The subcellular localization of mRNAs and miRNA family annotations were acquired from RNALocate [71] and TargetScan [72] databases, respectively. Additionally, TF-miRNA regulatory relationships were incorporated from the transmiR database [73].

### 4.10. Functional Enrichment Analysis

Metascape [74] was used to perform detailed annotations of various key factors, including gene symbols, synonyms, descriptions, functional annotations in canonical pathways, KEGG pathways, hallmark gene sets, and cellular localization information from GO, phenotype/genotype/disease information from DisGeNET, and their expression levels in common tissues. Enrichment analyses were also executed across multiple databases, including GO, KEGG, canonical pathways, hallmark gene sets, reactome gene sets, wikiPathways, CORUM, immunologic signatures, oncogenic signatures, TRRUST, and DisGeNET.

### 4.11. PPI Network Analysis and CMap Analysis

The R package “STRINGdb (v2.12.1)” with the STRING database v12.0 [33] was applied to construct PPI networks of various key biomarkers. The CMap database [34] was employed to explore the associations between key biomarkers and perturbation factors, so as to identify compounds suitable for the treatment of NADC in PLWH.

### 4.12. Implementation of NADCdb Web Interfaces

All processed data and analytical pipelines are hosted in NADCdb, a comprehensive and flexible platform constructed using HTML5, PHP7, CSS3, and JavaScript. We employed several external software packages to display data within NADCdb, including the Bootstrap v4.4.1 framework for organizing web interfaces, MySQL (v14.14) for backend storage and querying of processed data, DataTables (v1.10.12) for presenting tabular results, Highcharts (v5.0.0) for visualizing diverse results, and CGI/Perl5 (v16) for data analysis in the tool module.

## Figures and Tables

**Table 1:** Overview of diagnostic biomarkers identified by “dNADC” in 16 NADCs.

HIV Sample Type	Cancer	NO. of Key Genes Without LASSO	NO. of Key Genes with LASSO
non-ART	BLCA	78	3
non-ART	BRCA	106	21
non-ART	COAD	82	1
non-ART	ESCA	116	6
non-ART	HNSC	110	10
non-ART	KICH	100	5
non-ART	KIRC	115	15
non-ART	KIRP	95	9
non-ART	LIHC	76	13
non-ART	LUAD	89	12
non-ART	LUSC	109	4
non-ART	PRAD	49	5
non-ART	READ	95	5
non-ART	STAD	60	4
non-ART	THCA	65	11
non-ART	UCEC	115	6
ART	BLCA	5	3
ART	BRCA	6	4
ART	COAD	9	7
ART	ESCA	6	2
ART	HNSC	7	5
ART	KICH	6	3
ART	KIRC	8	3
ART	KIRP	8	2
ART	LIHC	7	5
ART	LUAD	6	5
ART	LUSC	9	3
ART	PRAD	6	3
ART	READ	8	6
ART	STAD	7	7
ART	THCA	7	3
ART	UCEC	6	1

**Table 2:** Overview of risk assessment indicators identified by “rNADC” in 21 NADCs.

Cancer	Key Factors	Clinical Markers
ACC	7	12
BLCA	43	25
BRCA	23	24
CESC	66	12
COAD	32	31
DLBC	35	12
ESCA	14	11
KICH	9	3
KIRC	16	7
KIRP	8	7
LIHC	37	12
LUAD	2	16
LUSC	6	8
OV	30	13
PRAD	24	11
SKCM	10	7
STAD	20	9
TGCT	89	7
THCA	3	5
UCEC	28	6
UCS	32	5

**Table 3:** Overview of immune biomarkers recognized by “iPredict” in 16 NADCs without ART.

Group	Cancer	Immune Biomarkers	Immune Biomarkers of Validated Cohort
CD4-	BLCA	313	/
CD4-	BRCA	364	/
CD4-	COAD	387	20
CD4-	ESCA	313	/
CD4-	HNSC	265	/
CD4-	KICH	479	171
CD4-	KIRC	137	41
CD4-	KIRP	198	57
CD4-	LIHC	299	/
CD4-	LUAD	357	44
CD4-	LUSC	538	42
CD4-	PRAD	215	/
CD4-	READ	342	17
CD4-	STAD	227	/
CD4-	THCA	206	/
CD4-	UCEC	478	/
CD8+	BLCA	242	/
CD8+	BRCA	145	/
CD8+	COAD	127	8
CD8+	ESCA	181	/
CD8+	HNSC	180	/
CD8+	KICH	133	32
CD8+	KIRC	190	88
CD8+	KIRP	153	59
CD8+	LIHC	173	/
CD8+	LUAD	106	11
CD8+	LUSC	134	13
CD8+	PRAD	56	/
CD8+	READ	107	8
CD8+	STAD	195	/
CD8+	THCA	67	/
CD8+	UCEC	94	/
CD4-CD8+	BLCA	143	/
CD4-CD8+	BRCA	247	/
CD4-CD8+	HNSC	178	/
CD4-CD8+	KIRC	123	29
CD4-CD8+	KIRP	76	19
CD4-CD8+	LIHC	223	/
CD4-CD8+	LUAD	229	24
CD4-CD8+	LUSC	319	24

Note: “/” indicates missing validation set data.

**Table 4:** Overview of immune biomarkers recognized by “iPredict” in 16 NADCs with ART.

Group	Cancer	Immune Biomarkers	Immune Biomarkers of Validated Cohort
CD4-	BLCA	93	/
CD4-	BRCA	152	/
CD4-	COAD	160	2
CD4-	ESCA	89	/
CD4-	HNSC	107	/
CD4-	KICH	241	83
CD4-	KIRC	63	24
CD4-	KIRP	66	22
CD4-	LIHC	121	/
CD4-	LUAD	152	11
CD4-	LUSC	242	11
CD4-	PRAD	98	/
CD4-	READ	141	3
CD4-	STAD	100	/
CD4-	THCA	84	/
CD4-	UCEC	191	/
CD8+	BLCA	91	/
CD8+	BRCA	58	/
CD8+	COAD	48	3
CD8+	ESCA	41	/
CD8+	HNSC	49	/
CD8+	KICH	88	21
CD8+	KIRC	72	33
CD8+	KIRP	52	27
CD8+	LIHC	72	/
CD8+	LUAD	57	5
CD8+	LUSC	97	6
CD8+	PRAD	25	/
CD8+	READ	41	2
CD8+	STAD	54	/
CD8+	THCA	19	/
CD8+	UCEC	24	/
CD4-CD8+	BLCA	51	/
CD4-CD8+	BRCA	117	/
CD4-CD8+	HNSC	68	/
CD4-CD8+	KIRC	61	18
CD4-CD8+	KIRP	23	10
CD4-CD8+	LIHC	116	/
CD4-CD8+	LUAD	117	10
CD4-CD8+	LUSC	185	10

Note: “/” indicates missing validation set data.

## Data Availability

The data and code supporting this study are available through the Download page of the NADCdb repository (http://bioinformaticsscience.cn/nadcdb/download.php, accessed on 1 January 2026).

## Supplementary Materials

The following supporting information can be downloaded at https://www.mdpi.com/article/10.3390/ijms27031169/s1.

## Abbreviations

The following abbreviations are used in this manuscript:

NADCs	Non-AIDS-defining cancers
PLWH	People living with HIV
DEGs	Differentially expressed genes
RF	Random Forest
CIT	Conditional Inference Trees
WGCNA	Weighted gene co-network analysis
PPI	Protein–protein interaction
CMap	Connectivity Map
ADC	AIDS-defining cancers
HAART	Highly active antiretroviral therapy
HACRC	HIV-1 associated colorectal cancer
HALC	HIV-1 associated lung cancer
HARC	HIV-1 associated renal cancer
KEGG	Kyoto Encyclopedia of Genes and Genomes
AUC	Area Under the Curve
LASSO	Least Absolute Shrinkage and Selection Operator
TFs	Transcription factors
miRNAs	MicroRNAs
GO	Gene ontology
ART	Antiretroviral therapy
non-ART	No ART
PBMC	Peripheral Blood Mononuclear Cells
ACC	Adrenocortical Carcinoma
BLCA	Bladder Urothelial Carcinoma
BRCA	Breast Invasive Carcinoma
CESC	Cervical Squamous Cell Carcinoma and Endocervical Adenocarcinoma
CHOL	Cholangiocarcinoma
COAD	Colon Adenocarcinoma
DLBC	Diffuse Large B-cell Lymphoma
ESCA	Esophageal Carcinoma
GBM	Glioblastoma Multiforme
HNSC	Head and Neck Squamous Cell Carcinoma
KICH	Kidney Chromophobe
KIRC	Kidney Renal Clear Cell Carcinoma
KIRP	Kidney Renal Papillary Cell Carcinoma
LAML	Acute Myeloid Leukemia
LGG	Brain Lower Grade Glioma
LIHC	Liver Hepatocellular Carcinoma
LUAD	Lung Adenocarcinoma
LUSC	Lung Squamous Cell Carcinoma
MESO	Mesothelioma
OV	Ovarian Serous Cystadenocarcinoma
PAAD	Pancreatic Adenocarcinoma
PCPG	Pheochromocytoma and Paraganglioma
PRAD	Prostate Adenocarcinoma
READ	Rectum Adenocarcinoma
SARC	Sarcoma
SKCM	Skin Cutaneous Melanoma
STAD	Stomach Adenocarcinoma
TGCT	Testicular Germ Cell Tumors
THCA	Thyroid Carcinoma
THYM	Thymoma
UCEC	Uterine Corpus Endometrial Carcinoma
UCS	Uterine Carcinosarcoma
UVM	Uveal Melanoma
TCGA	The Cancer Genome Atlas
GTEx	Genotype-Tissue Expression
FDR	False discovery rate
TPM	Transcripts per million
