# Supplementary material for: NADCdb: A Joint Transcriptomic Database for Non-AIDS-Defining Cancer Research in HIV-Positive Individuals
Source: Int J Mol Sci. 2026 Jan 23;27(3):1169. doi: 10.3390/ijms27031169 (PMC12897083; doi:10.3390/ijms27031169)
Supplement: Supplementary file 1 [file ijms-27-01169-s001.zip › ijms-4019593-supplementary.pdf]

Supplemental Materials

NADCdb: A Joint Transcriptomic Database for Non-AIDS-Defining Cancer Research in HIV-Positive Individuals

Table S1 Sample sizes of PLWH cohorts analyzed

Accession number	Sample source	HIV free	HIV_nonART	HIV_ART	Technology
GSE2171	PBMC	12	22	18	Microarray
GSE10924	PBMC	48	0	48	Microarray
GSE4124	PBMC	20	25	0	Microarray
GSE44228	PBMC	33	36	36	Microarray
GSE33877	PBMC	6	6	0	Microarray
GSE125223	PBMC	4	5	8	RNA-seq
GSE199911	PBMC	24	0	0	RNA-seq
GSE185027	PBMC	0	0	5	RNA-seq
GSE77939	Whole blood	4	5	12	Microarray
GSE57730	Whole blood	12	12	0	Microarray
GSE71063	Whole blood	20	20	0	Microarray
GSE71064	Whole blood	20	0	20	Microarray
GSE200606	Whole blood	8	8	0	Microarray
GSE56837	Whole blood	26	26	0	Microarray
GSE155352	Whole blood	4	40	0	RNA-seq

Table S2 Sample sizes of cancer cohorts analyzed

Cancer	TCGA_tumor	TCGA_normal[a]	GTEx[b]
ACC	77	0	126
BLCA	408	19	9
BRCA	1095	99	178
CESC	303	0	10
COAD	458	41	307
DLBC	47	0	337
ESCA	184	13	271
HNSC	515	44	0
KICH	66	25	28
KIRC	533	72	28
KIRP	290	32	28
LIHC	371	49	110
LUAD	516	58	288
LUSC	501	51	288
OV	420	0	88
PRAD	497	51	100
READ	163	10	0
SKCM	102	0	323

STAD	416	36	174
TGCT	148	0	165
THCA	120	57	337
UCEC	1043	35	279
UCS	57	0	78

[a]The TCGA_normal dataset was used as the cancer control cohort for the dNADC module.

[b]The GTEx dataset was used as the cancer control cohort for the rNADC module.

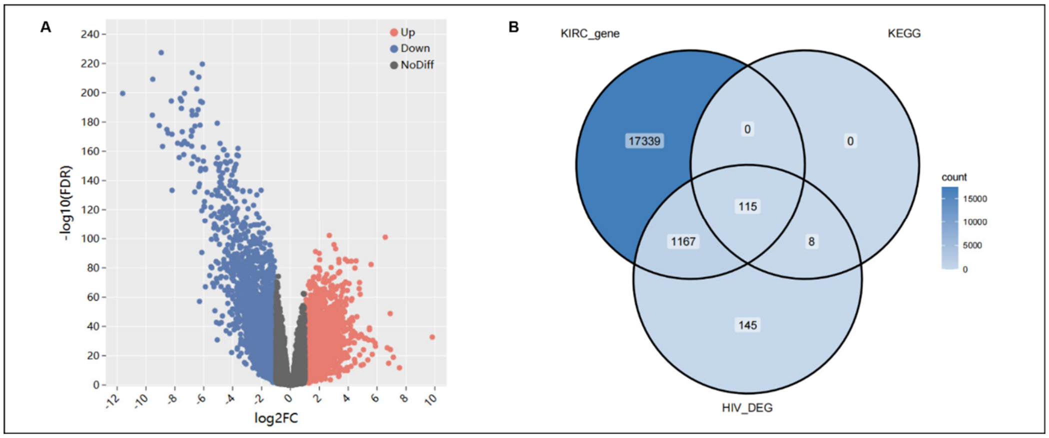

Figure S1. Key genes for HARC. (A) Volcano plot of differentially expressed genes. (B) Venn diagram depicting the intersection of KIRC genes, KEGG-derived differential/upstream genes, and HIV-associated differentially expressed genes.

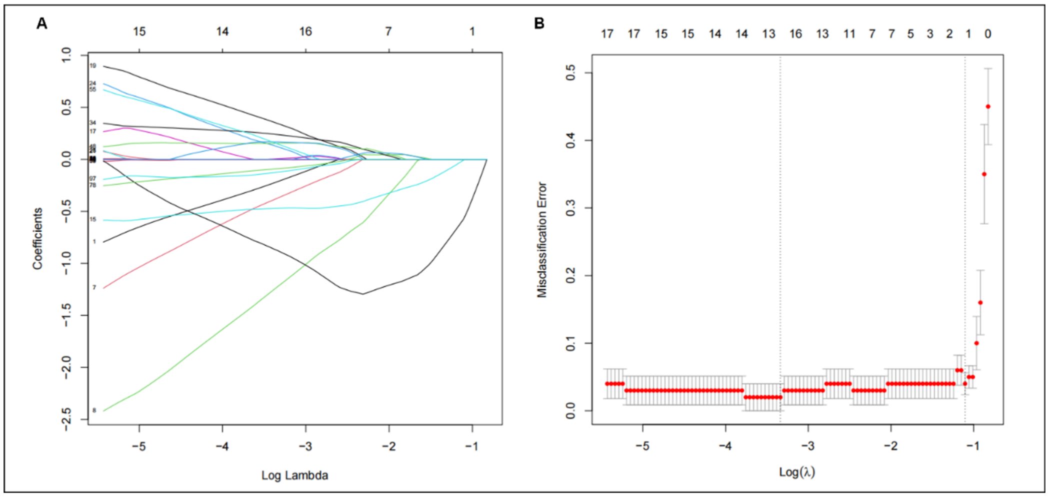

Figure S2. Machine learning-based establishment of HARC diagnostic biomarkers and construction of predictive models. (A-B) Coefficient profile plot from Lasso Regression Feature Selection.
